# E26 transformation-specific transcription variant 5 in development and cancer: modification, regulation and function

**DOI:** 10.1186/s12929-023-00909-3

**Published:** 2023-03-06

**Authors:** Yi Wei, Shenqi Han, Jingyuan Wen, Jingyu Liao, Junnan Liang, Jingjing Yu, Xiaoping Chen, Shuai Xiang, Zhao Huang, Bixiang Zhang

**Affiliations:** 1 Hepatic Surgery Center, Tongji Hospital, Tongji Medical College, Huazhong University of Science and Technology, Wuhan, China; 2 Clinical Medical Research Center of Hepatic Surgery at Hubei Province, Wuhan, China; 3 Hubei Key Laboratory of Hepato-Pancreatic-Biliary Diseases, Tongji Hospital, Tongji Medical College, Huazhong University of Science and Technology, Wuhan, China; 4 Key Laboratory of Organ Transplantation, Ministry of Education, Wuhan, China; 5 Key Laboratory of Organ Transplantation, National Health Commission, Wuhan, China; 6 Key Laboratory of Organ Transplantation, Chinese Academy of Medical Sciences, Wuhan, China

**Keywords:** ETV5, Post-translational modification, Immune, Angiogenesis, Metastasis, Cell cycle, Drug target

## Abstract

E26 transformation-specific (ETS) transcription variant 5 (ETV5), also known as ETS-related molecule (ERM), exerts versatile functions in normal physiological processes, including branching morphogenesis, neural system development, fertility, embryonic development, immune regulation, and cell metabolism. In addition, ETV5 is repeatedly found to be overexpressed in multiple malignant tumors, where it is involved in cancer progression as an oncogenic transcription factor. Its roles in cancer metastasis, proliferation, oxidative stress response and drug resistance indicate that it is a potential prognostic biomarker, as well as a therapeutic target for cancer treatment. Post-translational modifications, gene fusion events, sophisticated cellular signaling crosstalk and non-coding RNAs contribute to the dysregulation and abnormal activities of ETV5. However, few studies to date systematically summarized the role and molecular mechanisms of ETV5 in benign diseases and in oncogenic progression. In this review, we specify the molecular structure and post-translational modifications of ETV5. In addition, its critical roles in benign and malignant diseases are summarized to draw a panorama for specialists and clinicians. The updated molecular mechanisms of ETV5 in cancer biology and tumor progression are delineated. Finally, we prospect the further direction of ETV5 research in oncology and its potential translational applications in the clinic.

## Background

The E26 transformation-specific (ETS) family is an evolutionarily conserved transcription factor family represented by 28 protein-coding genes in the human genome [1]. The unifying feature of ETS family proteins is the ETS DNA-binding domain (DBD), which displays three conserved α-helixes and a four-stranded antiparallel β-sheet that together form a winged helix-turn-helix motif [2, 3]. ETS DBD recognizes up to nine consecutive bases consisting of the central core sequence GGA(A/T), three bases located upstream and three bases located downstream of the GGA sequence, which contributes to the different affinities in interacting with specific DNA sites [4]. Based on their differences in binding preference, ETS transcription factors are classified into four different classes, named class I to IV.

As a member of Class I, ETV5 is characterized by low affinity to C in the third base upstream of the GGA motif [4, 5]. It emerged as a critical oncogenic transcriptional factor in various cancers and has drawn extensive attention in past decades. ETV5 is expressed ubiquitously in human tissues, with high-level expression observed in the testis, lung and brain, in agreement with the essential roles of ETV5 during development [6–8]. Under normal physiological conditions, the expression of ETV5 is tightly controlled by sophisticated transcriptional mechanisms and post-translational modifications. Gene fusion events, dysfunction of cell signaling, as well as dysregulation at the post-transcriptional and post-translational levels, all contribute to the pathological expression of ETV5 [9–11]. Functionally, ETV5 exerts important effects on branching morphogenesis, neural system development, fertility, energy metabolism and the immune response [7, 12–14]. ETV5 also plays critical roles in the formation and development of various cancers, affecting virtually all hallmarks of cancer cells, including the epithelial–mesenchymal transition (EMT), angiogenesis, cell cycle, oxidative stress and drug resistance [15–19]. ETV5 displayed higher expression in many types of cancers compared to matched normal tissues, and its expression was a predictor of poor survival [20–26]. As a consequence, significant efforts have been made to elucidate the roles of ETV5 in health and in pathological processes.

In this review, we specified the molecular structure, functional modules and post-translational modifications of ETV5. In addition, its critical roles in maintaining organ homeostasis and relevant molecular mechanisms are also introduced. As a high-profile oncogenic transcriptional factor, its expression patterns and clinical value in various cancers are summarized and discussed. Finally, we delineated the effects of ETV5 on multiple hallmarks of cancer cells, and proposed it as a potential diagnostic biomarker, as well as a therapeutic target, for further translational research.

## Structure of ETV5

**Fig. 1:**
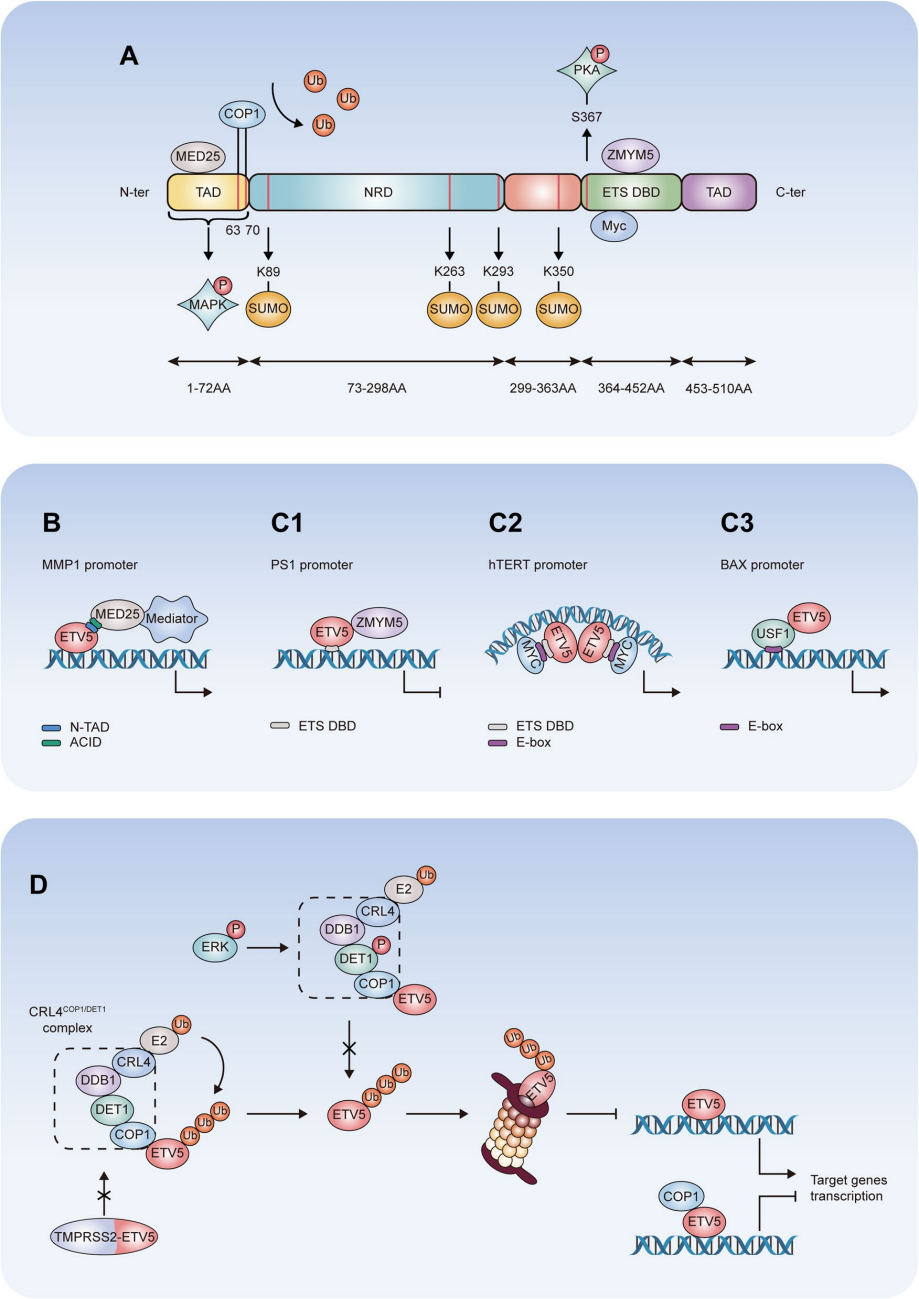
The structure and protein-protein interactions of ETV5. **A** ETV5 contains four functional domains: N-terminal (N-ter) TAD, NRD, ETS, DBD and C-terminal (C-ter) TAD. ETV5 is subjected to modification at the post-translational level by phosphorylation, SUMOylation and ubiquitylation. The sites of PKA-dependent phosphorylation and SUMOylation, as well as protein binding are marked. Note that the phosphorylation and ubiquitylation sites have not been identified. **B** ETV5 interacts with Mediator complex subunit 25 (MED25) as a coactivator and recruits cofactor mediator of RNA polymerase II transcription (Mediator) to the MMP1 promoter. **C1**,** C2**,** C3** ETV5 suppresses PS1 transcription by interacting with zinc finger MYM-type containing 5 (ZMYM5). ETV5 interacts with E-box of MYC proto-oncogene (MYC) symmetrically on human TERTp promoter. ETV5 promotes BAX transcription by interacting with upstream transcription factor 1 (USF1) without binding the promoter. **D** Formation of the CRL4^COP1/DET1^ complex leads to proteasome-mediated degradation of ETV5 protein. Fusion of ETV5 with TMPRSS2 and phosphorylation of DET1 inhibits this degradation pathway of ETV5. COP1 interacts with ETV5 to suppresses its transcription activity

The gene encoding ETV5 is located on human chromosome 3 in the region q27.2. It encodes a 57.8 kDa protein containing four functional domains: a PEA3 subfamily characteristic amino-terminal acidic transactivation domain (TAD), a central negative regulatory domain (NRD), a conserved ETS DNA-binding domain (DBD), and a carboxy-terminal TAD (Fig. 1A) [27].

The first 72 amino acid (AA) residues constitute the N-terminal TAD, and residues 452–510 AA constitute the C-terminal TAD. The N-terminal TAD contains two functional constitutive photomorphogenic 1 (COP1)-interacting sites at positions 63 and 70, which lead to the ubiquitylation and proteasomal degradation of ETV5 (Fig. 1A). The N-terminal TAD mediates transcriptional activation through the interaction with the cofactor mediator of RNA polymerase II transcription [28]. Mediator complex subunit 25 physically associates with N-terminal TAD of ETV5 as a coactivator and recruits Mediator to the promoter of target gene matrix metallopeptidase (MMP) 1 to initiate transcription by RNA polymerase II (Fig. 1B) [27, 29]. NRD, mapped from residues 73 to 298 AA, inhibits the transcriptional activity of N-terminal TAD, which is dependent on the its small ubiquitin-related modifier (SUMO) sites (K89, K263 and K293) (Fig. 1A) [30].

The ETS DBD (363 to 451 AA) of ETV5 recognizes the 5′-GGA(A/T)-3′ motif in gene promoter regions, and is characterized by low affinity to C in the third base upstream of the GGA motif. This domain contains a phosphorylation site (Ser367) that can be transactivated by the protein kinase A (PKA) pathway [31]. The protein binding patterners of ETV5 modulate the transcriptional activity of its ETS DBD. ETV5 was found to promote Presenilin 1 (PS1) transcription by the interaction of ETS DBD with the PS1 promoter. Zinc finger MYM-type containing 5 was found to interact with ETV5 bound to the PS1 promoter and form an inhibitory complex with the ETV5-PS1 promoter complex, which repressed PS1 transcription (Fig. 1C1) [32]. ETS DBD interacts with the E-box of MYC proto-oncogene and forms two identical composite ETS/E-box motifs symmetrically surrounding the telomerase reverse transcriptase promoter (TERTp) to promote its transcription (Fig. 1C2) [33, 34]. ETV5 promotes BCL2 associated X (BAX) transcription by interacting with the E-box of upstream transcription factor 1 to form a ternary complex on the BAX promoter, instead of directly binding to the promoter (Fig. 1C3) [35]. At the same time, both the N- and C-terminal amino acid residues of the ETS domain can inhibit DNA binding independently, but also act cooperatively to yield higher than additive levels of inhibition [36]. The C-terminal inhibitory domain (CID) and the N-terminal inhibitory domain (NID) acts through a distinct mechanism. CID disturbs the relative position of its DNA-recognition helix. NID is intrinsically disordered and inhibits the function of ETS DBD through interaction with the CID or helix H3 [36].

## Post-translational modification of ETV5

Protein phosphorylation is the most common post-translational modification involved in the regulation of protein activity and metabolism. Rapidly reversible phosphorylation is a ubiquitously utilized mechanism for responding to extracellular signals and affecting the activity of transcriptional factors (TFs) [37]. ETV5 contains three potential PKA phosphorylation sites, Ser367, Ser242 or/and Ser248, the former of which is located at the beginning of the ETS DBD (Fig. 1A). After incubation with PKA, only mutations of Ser367 resulted in decreased transcriptional activation [31]. PKA-mediated phosphorylation of Ser367 changes the conformation and decreases the DNA binding activity of ETV5, which subsequently increases its transcriptional activity [31]. Mitogen-activated protein kinase (MAPK) signaling also participates in the phosphorylation of ETV5, but the precise mechanism and the MAPK-dependent phosphorylation position remains unknown [38].

SUMOylation is a reversible post-translational modification of K residues that affect the stability, activity, and localization of many TFs, including those of the ETS family [39]. ETV5 can be modified with SUMO at multiple sites including K89, K263, K293 and K350 (Fig. 1A), resulting in multiple SUMO-modified forms of ETV5 in cells [40, 41]. SUMOylation at K89, K263 and K293 in the NRD reduces the transcriptional activity of ETV5, but it does not affect its subcellular localization, DNA-binding capacity, or stability [30]. SUMOylation at one of the three binding sites is sufficient to repress transcription, but the presence of all three SUMO sites is required for the maximal inhibition of the NRD [30]. Mutation of NRD SUMOylation sites did not completely abrogate the suppression of the activity of full-length ETV5, as the site K350 outside of the NRD can also be modified with SUMO to repress transcription [40]. Although many studies demonstrated that SUMOylation affects the transcriptional activity of ETV5, the specific mechanism of this effect remains unclear.

The proteasomal degradation system promotes the rapid elimination of TFs and facilitates recruitment of non-phosphorylated TFs to additional rounds of transcription [37]. High levels of ETV5 mRNA are detected in human ovarian cancer, but the protein is almost undetectable in the corresponding tissues [23]. Therefore, highly efficient degradation of ETV5 protein was postulated. It has been demonstrated that ETV5, like many other TFs, is a highly unstable protein with a half-life of 30–60 min, and undergoes degradation via the 26S proteasome pathway [42]. COP1 is a ubiquitously expressed E3 ubiquitin ligase which can indirectly induce the degradation ETV5 by recruiting de-etiolated 1 (DET1) or repress the transcriptional activity of ETV5 [43, 44]. COP1, DET1, and DNA damage-binding protein 1 (DDB1) interact with Cul4-RING ubiquitin ligase (CRL4) to form a CRL4^COP1/DET1^ complex, which mediates the degradation of ETV5 [9, 14]. In the presence of COP1 and DET1, COP1 acts as a linker between ETV5 and DET1 through two functional COP1-interacting sites in the N-terminal part of ETV5, leading to the ubiquitylation and proteasome-mediated degradation of ETV5 [45]. When the COP1 level exceeds that of DET1, ETV5 protein is not destabilized but its transcriptional activity is reduced, suggesting a DET1-independent function of COP1 in the regulation of ETV5 transcriptional activity [45]. Tumors driven by Kras^d12^ have higher levels of ETV5 protein than matched normal tissues, but this is not caused by an increase of the mRNA level [13]. Cancer cells with activated Ras signaling stabilize ETV5 protein through enhanced DET1 phosphorylation at Ser458, which inhibits the degradation of ETV5 [13]. Gene fusion of ETV5 with transmembrane protease serine 2 (TMPRSS2) also increases the protein stability of ETV5 by disrupting COP1-binding sites within the N-TAD (Fig. 1D) [1].

## ETV5 in physiological activities

### Branching morphogenesis

**Fig. 2:**
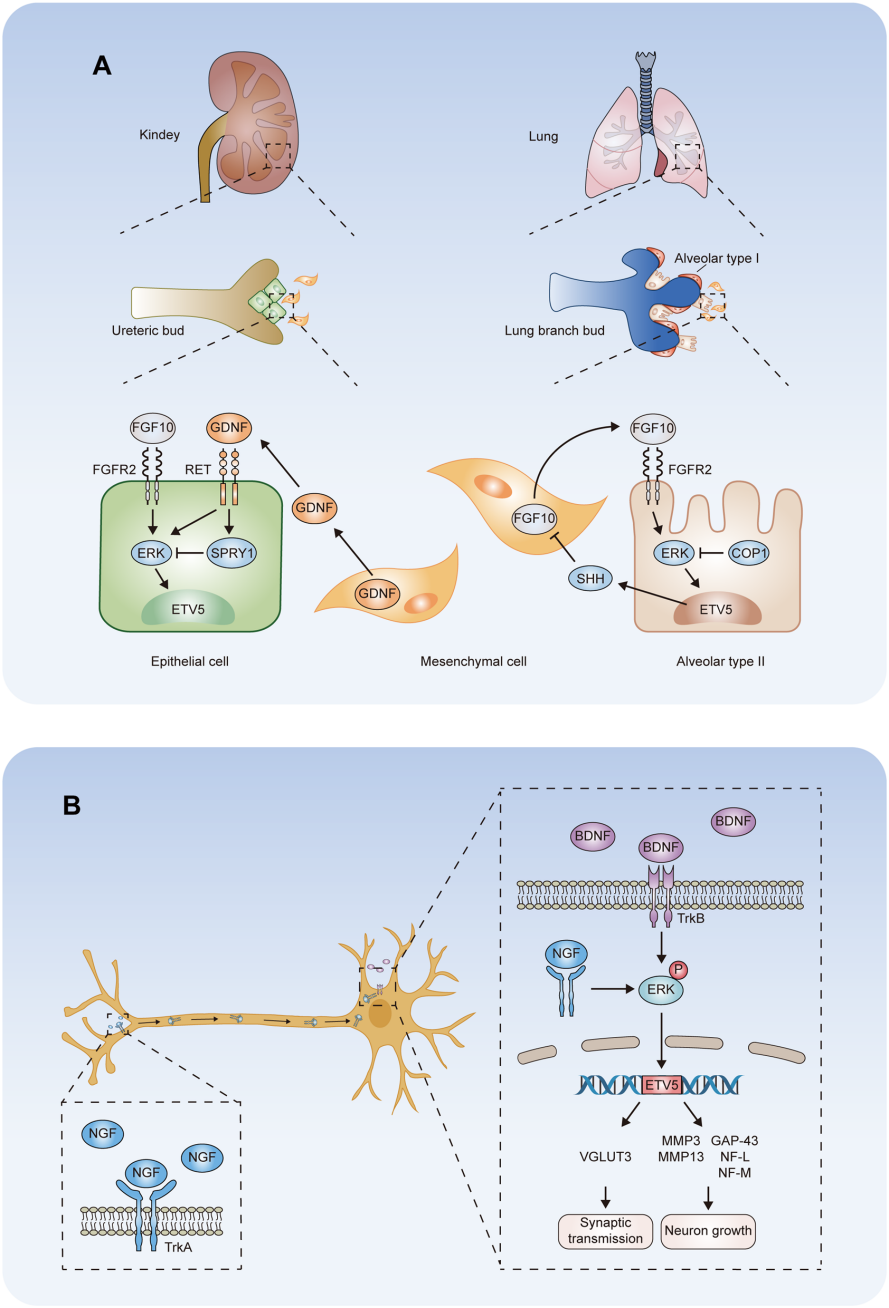
ETV5 in branching morphogenesis and neural system development. **A** ETV5 contributes to the formation of the ureteric bud. GDNF and FGF10 signaling activate the ERK/MAPK cascade and positively regulate ETV5 expression in epithelial cells. SPRY1 acts as a negative regulator of receptor tyrosine kinases and reduces the effect of GDNF signaling. ETV5 modulates lung branching bud growth through the regulation of FGF10-SHH signaling and COP1 in alveolar type II cells and adjacent mesenchyme. **B** In neurons, NGF-TrkA and BDNF-TrkB complexes activate ETV5 in an ERK/MAPK-dependent mechanism. ETV5 promotes the transcription of vesicular glutamate transporter 3 (VGLUT3), MMP3, MMP13, growth-associated protein (GAP-43), medium neurofilament (NF-M) and light neurofilament (NF-L), contributing to synaptic transmission and neuronal growth

ETV5 promotes normal kidney development by mediating the formation of the ureteric bud (UB) tip domain and inducing directed cell movements [46, 47]. Single-cell transcriptomics in embryonic mouse kidneys revealed extensive variation of ETV5 expression during kidney development and demonstrated that ETV5 acts as a downstream target of activated ret proto-oncogene (RET) [12]. During UB development, ETV5 expression is positively regulated by glial cell line derived neurotrophic factor (GDNF) and fibroblast growth factor (FGF) 10 signaling [48, 49]. In GDNF^−/−^ transgenic mouse, ETV5 expression is significantly reduced along with dysregulation of the downstream genes C-X-C motif chemokine receptor 4 (CXCR4) and MMP14, which induces irregular branch formation in hypoplastic kidneys [47]. In the absence of GDNF or Sprouty1, a well-known negative regulator of receptor tyrosine kinases, FGF10 can upregulate ETV5 and rescue the GDNF^−/−^ phenotype during UB development (Fig. 2A, left) [50, 51]. Conditional knockout of sex-determining gene SRY-box9 (SOX9) results in reduced expression of ETV5 and other RET downstream targets in ureteric tips, leading to hypoplastic kidneys and renal agenesis [52].

The mammalian lung is a highly branched network, in which the distal region of the bronchial tree transforms into alveolar air sacs during development [53]. The alveolar epithelium consists of alveolar type I (ATI) and alveolar type II (ATII) cells. Lung injury activates the stem-cell function of ATII to create new ATII cells and differentiate into ATI cells [53]. ETV5 deletion in ATII cells induces the expression of signature genes of ATI cells and impairs lung recovery following bleomycin-induced injury, which indicate that ETV5 is required to maintain the identity of ATII cells in the adult lung [13]. ETV5 gene expresses was found to be exclusively activated in the distal epithelium of the developing mouse lung, and the expression profile was strongly correlated with the spatial distribution of ATII cells [54, 55]. The ubiquitin proteasome system also functions in lung development. Inactivation of COP1 in developing lung epithelium can stabilize ETV5 protein levels, leading to a delay of branching growth [56]. In lung epithelium, FGF1 and FGF7 act as mesenchymal signals to induce ETV5 expression in tracheal epithelium [57]. FGF10/fibroblast growth factor receptor 2 (FGFR2) activates the ERK/MAPK cascade and increases the activity of ETV5, which promotes sonic hedgehog signaling molecule (SHH) expression by directly interacting with the enhancer of SHH [6]. SHH in turn downregulates FGF10 in adjacent mesenchyme and redirects branch bud growth (Fig. 2A, right) [6]. Additionally, in SOX9 positive lung progenitor cells, the gene regulatory network implies that ETV5 acts as one of the central TFs in the maintenance and differentiation of distal tip epithelial cells [58].

### Neural system development

Neurons interact through the major neurotransmitters, γ-aminobutyric acid (GABA) and glutamate, which deliver excitatory and inhibitory signals [59]. ETV5 suppresses neurogenin 2 (NEUROG2) transcription by binding to its promoter with a transcriptional corepressor in neural progenitor cells (NPCs) [60]. During NPC differentiation toward neurons, ETV5 blocks the differentiation of glutamatergic neurons and increases the abundance of GABAergic neurons through the negative regulation of NEUROG2 [60]. ETV5 also plays essential roles in sensory neuronal growth and differentiation. During neural system development, dorsal root ganglion (DRG) neurons extend axons over a long distance to perceive stimuli from target cells [61]. Nerve growth factor (NGF), a well-studied target-derived neurotrophin, activates tropomyosin receptor kinase (Trk) A in the distant axons [62]. In developing DRG neurons, the NGF-TrkA complex is subjected to retrograde transport from axons to the cell body, where it activates the MAPK pathway, thereby significantly inducting ETV5 transcription [63]. Elevated ETV5 levels are necessary for sensory neuron axonal growth and neuronal differentiation via the upregulation of MMP3 and MMP13 in an NGF-depended manner (Fig. 2B) [63]. Brain-derived neurotrophic factor (BDNF) and its cognate receptor TrkB are critical for the survival and synapse formation of sensory neurons, as well as synaptic transmission [64]. In DRG neurons, BDNF upregulates ETV5 expression in a ERK1/2-dependent manner [7]. ETV5 is necessary for BDNF-induced synaptic transmission and neurite outgrowth by promoting vesicular glutamate transporter 3, growth-associated protein, medium neurofilament and light neurofilament expression (Fig. 2B) [7]. In hippocampal neurons, ETV5 is required for BDNF-mediated dendritic arbor development and spine formation [65]. Recently, a transcriptomic profile study of PEA3 subfamily members revealed that BDNF is significantly activated by ETV5 in hypothalamic cell lines [66]. During dendrite development, overexpression of Capicua transcriptional repressor (CIC) induces ETV5 deregulation, which significantly inhibits the number of dendritic tips and reduces the total dendrite length [67]. During brain development, post-transcriptional modification is active in neuronal stem cells. Ras signaling inactivates CRL4^COP1/DET1^, which induces the accumulation of ETV5 and Jun proto-oncogene [68]. The stabilization of ETV5 in COP1-knockout cells causes a lethal phenotype of newborn mice and promotes the expression of genes associated with glial cell development [68]. During glial development, ETV5 is regulated by MEK in deep cortical layers and contributes to glial progenitor specification and mature astrocyte differentiation [69].

### Fertility and embryonic development

ETV5 is important for fertility in both males and females, including spermatogenesis and embryonic survival. Spermatogenesis is a complex process, which proceeds through the mitotic division of spermatogonial stem cells (SSCs), meiotic cell division, homologous chromosome pair formation and the formation of haploid spermatids [70].

**Fig. 3:**
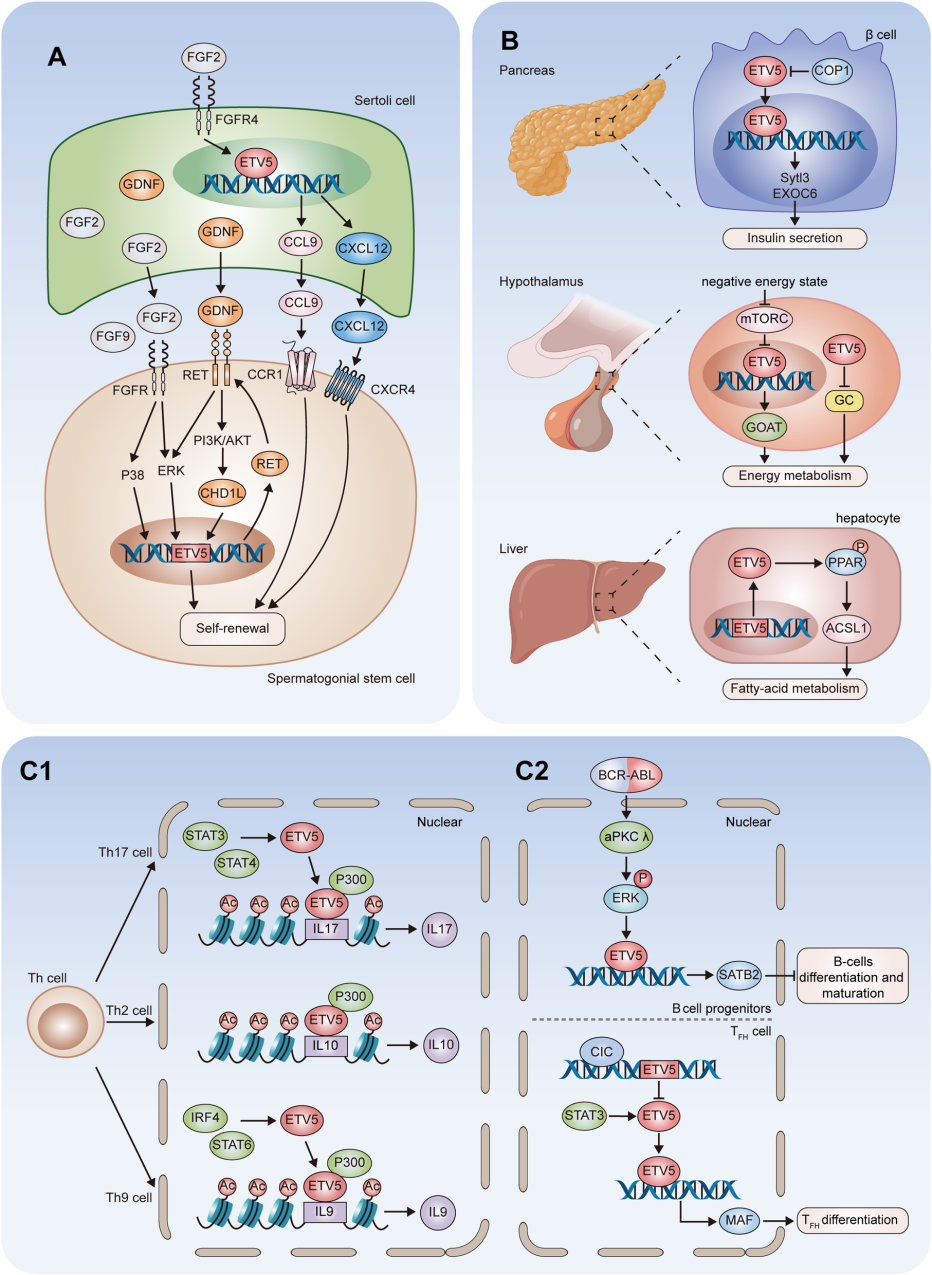
ETV5 in fertility and embryonic development, metabolic processes, and the immune system. **A** In Sertoli cells, FGF2 induces CXCL12 expression via the transcriptional regulation of ETV5. CXCL12, CCL9, GDNF and FGF2 released from Sertoli cells interact with receptors located on spermatogonial stem cells (SSCs) and activate intracellular signal cascades, which promote ETV5 expression and contribute to the self-renewal of SSCs. In addition, activated ETV5 promotes the transcription of the GDNF receptor RET. **B** ETV5 regulates insulin secretion and fatty acid metabolism in pancreatic beta cells and hepatocytes. ETV5 also regulated the energy balance in the brain. **C1** In Th17 cells, ETV5 is upregulated by STAT3 and STAT4. Elevated ETV5 levels promote IL17 production by binding to its promoter and recruiting histone acetyltransferase p300. In Th2 cells, ETV5 promotes IL10 expression. In Th9 cells, the upregulation of ETV5 by STAT6 and interferon regulatory factor 4 (IRF4) facilitates IL9 secretion. **C2** In T_FH_ cells, overexpressed ETV5 induced by CIC activates MAF expression in STAT3-dependent mechanism, contributing to T_FH_ cells differentiation. In B-cell progenitors, BCR-ALB enhanced aPKC λ activates ERK and ETV5, which transcriptionally upregulates SATB homoebox 2 (SATB2) to inhibit B-cell differentiation and maturation. *CHD1L* chromodomain helicase DNA binding protein 1 like and DEAD-box helicase 5, *CCR1* C-C-receptor type 1, *SYTL3* synaptotagmin-like 3, *EXOC6* exocyst-6, *GC* glucocorticoids, *ACSL1* acyl-CoA synthetase long chain family member 1

During spermatogenesis, ETV5 is regulated by chromodomain helicase DNA binding protein 1 like and DEAD-box helicase 5 in GDNF-dependent mechanism, which is essential for the self-renewal of SSCs (Fig. 3A) [71, 72]. GDNF/RET signaling activates the ERK/MAPK cascade and increases the expression of ETV5 and BCL6B transcription repressor (BCL6B), which contributes to continuous proliferation of germ cells and promotes the self-renewal of SSCs [73]. ETV5^−/−^ mice exhibit decreased RET expression and disrupted SSC self-renewal, which may be caused by an impairment of GDNF/RET signaling [74]. FGF9 and FGF2 induce p38/MAPK phosphorylation and the ERK/MAPK cascade, which in turn activates ETV5, increasing BCL6B expression, ultimately promoting the proliferation of SSCs [73, 75].

Sertoli cells are the only somatic cells within seminiferous tubules and provide the immediate environment for developing germ cells [70]. It was reported that ETV5 is expressed exclusively within Sertoli cells and altered the stem cell niche supported by Sertoli cells in the testis of mice [76]. During sex development, SOX9 upregulates ETV5 expression by binding with the upstream 5′ regulatory region of ETV5 in Sertoli cells of the XY gonad [77]. ETV5 expressed in Sertoli cells contributes to the integrity of the blood-testis barrier and immune response in the testes [78]. ETV5^−/−^ mice exhibit a Sertoli cell-only phenotype, with a lack of spermatogenic cells in the testes and no sperm in the epididymis [79]. Missense mutations of ETV5 also lead to a Sertoli cell only phenotype and increased embryonic lethality through the regulation of CXCR4 and C-C motif chemokine ligand 9 (CCL9), which are involved in the maintenance SSCs [80]. In Sertoli cells, ETV5 upregulates the mRNA expression of CCL9, which interacts with C-C-receptor type 1 located on SSCs and is responsible for the maintenance of SSCs [81]. After FGF2 treatment, stromal cell-derived factor 1 (SDF-1), also named C-X-C motif chemokine ligand 12 (CXCL12), is upregulated by ETV5 through the direct interaction with ETV5 and SDF-1 promoter in Sertoli cells [82]. CXCL12 interacts with its primary receptor CXCR4 expressed in SSCs, which contributes to the self-renewal and maintenance of SSCs (Fig. 3A) [82].

ETV5 is specifically expressed in undifferentiated mouse embryonic stem cells (ESCs), which implies a regulatory role of ETV5 in the proliferation and differentiation of ESCs [83]. ETV5 supports the self-renewal of ESCs when ERK signaling is inhibited, whereas when the ERK pathway is active, ETV5 facilitates the transition from naive pluripotency to formative pluripotency [84]. ETV5 was also found to contribute to the maintenance and reprogramming of mouse ESCs by positively regulating the expression of methylcytosine dioxygenase 2 [85]. In addition, ETV5 may orchestrate the specification of primitive endoderm and epiblast during the differentiation of mouse ESCs by upregulating GATA binding protein 6 (GATA6) and inhibiting FGF5, which eventually directs cells to a primitive endoderm fate [85]. A transcriptomic study revealed that ETV5 was directly regulated by GATA6 in human embryonic stem cells and may play a role in the maintenance of pluripotency [86].

### Metabolic processes

ETV5 is an obesity-related gene which plays important roles in the regulation of energy balance and metabolism (Fig. 3B). It has been demonstrated that a total loss of function of ETV5 results in reduced diet-induced obesity and severe glucose intolerance [87]. Genome-wide association studies have revealed an association of ETV5 with human obesity in multiple populations [88, 89]. ETV5^−/−^ mice are lean, but suffer from dysregulation of insulin exocytosis in pancreatic β cells [90]. ETV5 negatively regulates insulin secretion in β cells through transcriptional regulation of Synaptotagmin-like 3 and Exocyst-6 [44]. In the pancreas, ETV5 is one of the critical substrates of CRL4^COP1/DET1^, which downregulates ETV5 post-transcriptionally [14]. Elevated ETV5 levels impair insulin secretion and lead to glucose intolerance, indicating the key role of the CRL4^COP1/DET1^-ETV5 axis in nutrient-induced insulin secretion (Fig. 3B, upper) [14].

As a reaction to different nutrient states, expression of ETV5 changes in specific brain areas of adult rats, suggesting that ETV5 functions as an obesity-associated gene in the brain [91]. In response to stress, the hypothalamic-pituitary-adrenocortical (HPA) axis affects metabolism by inducing gluconeogenesis and hyperglycemia. It was also reported that ETV5 regulates the activity of the HPA axis by elevating plasma glucocorticoid levels [92]. In hypothalamic cells, ETV5 induces the expression of ghrelin-*O*-acyl-transferase (GOAT), which regulates multiple important metabolic functions. ETV5 was found to increase the transcriptional activity of the GOAT gene promoter in an mTORC1-dependent manner (Fig. 3B, middle) [93].

ETV5^−/−^ hepatocytes exhibited increased lipid accumulation, indicating a role of ETV5 in fatty acid metabolism [94]. Peroxisome proliferator-activated receptors (PPARs) are fatty acid-activated nuclear receptors that mediate energy metabolism via the interaction with PPAR elements (PPREs) in the promoters of target genes [95]. ETV5 markedly enhanced PPRE trans-activity through S248 phosphorylation and promoted the mRNA expression of the PPAR downstream gene acyl-CoA synthetase long chain family member 1 (Fig. 3B, lower) [94].

### Immune system

Growing evidences elucidate the crucial roles of ETV5 in immune modulation. In the model of allergic airway inflammation, mice with conditional knockout of ETV5 in T cells display reduced allergic airway inflammation through the decreased interleukin (IL)-17 production in lung tissues [96]. Mice with specific-T cells ETV5-deficiency exhibit less mast cells accumulation and polymorphonuclear leukocyte infiltrations through the decreased production of IL-9 in OVA-induced allergic airway inflammation animal model [97]. Tissue-resident memory T cells (Trm) cells are a subset of CD8^+^ T cells which positions within nonlymphoid tissues without entering the peripheral blood [98]. In liver-specific CIC-knockout mice, the upregulation of ETV5 induces liver injury via the transcriptional regulation of homolog of Blimp-1 in T cell, which promotes CD8^+^ Trm cell development in livers [99]. γδ T cells constitute 4% of CD3^+^ T cells in the human peripheral blood with the CD4^−^/CD8^−^ phenotype [100]. ETV5 induces IL17 transcription in mature thymocyte and regulates γδ T cells differentiation through promoting the maturation of IL-17-producing γδ effector cells [101].

CD4^+^ T helper (Th) cells are essential components of the immune system. Following cytokine stimulation, activated signal transducer and activator of transcription (STAT) family proteins drive the differentiation of Th cells into distinct functional subsets [102]. In Th17 cells, stimulated by IL-6 and IL-23, STAT3 directly binds to the ETV5 promoter to activate ETV5 expression, which further recruits histone acetyltransferase p300 at the IL-17 locus and promotes the production of IL-17 in Th17 cells (Fig. 3C1, upper) [96]. In the experimental colitis models, ETV5 induces severe intestinal mucosal inflammation through the increased Th1/Th17 immune response [103]. Overexpression of ETV5 induced by STAT3/STAT4 activation promotes Th1/Th17 differentiation and IL-17 A production, which contributes to intestinal inflammation in inflammatory bowel diseases [103]. During human Th1/Th2 commitment, IL-12 promotes ETV5 expression through the activation of STAT4 which induced selectively high levels of ETV5 in activated Th1 CD4^+^ cells compared with the naive and memory Th1 cells [104, 105]. In an Aspergillus fumigatus extract-induced inflammation model, mice with T cell-specific ETV5-knockout have decreased population of IL-10-producing Th cells [106]. ETV5 increases IL-10 secretion in Th2 cells by directly binding to the IL-10 locus and facilitating the interaction of other IL-10-induced TFs (Fig. 3C1, middle) [106]. STAT6/interferon regulatory factor 4 elevate ETV5 expression, which binds to IL-9 locus and recruits histone acetyltransferases to promote IL-9 production in Th9 cells (Fig. 3C1, lower) [97]. Conditional knockout of CIC in hematopoietic lineage cells induced upregulated ETV5 expression and abnormal proliferation of follicular helper CD4^+^ T (T_FH_) cells, which are essential for the formation of germinal centers and the maintenance of B cells differentiation [107, 108]. During T_FH_ cells development, overexpressed ETV5 interacts with the MAF promoter and transcriptionally activates MAF expression in STAT3-dependent mechanism, which contribute to the abnormalities of T_FH_ cells differentiation in CIC-knockout mice (Fig. 3C2, lower) [108].

In B-cell progenitors, BCR-ALB oncogene enhanced atypical protein kinase C (aPKC) λ expression, both of which are critical to the progression of B-cell acute lymphoblastic leukemia [109]. The aPKC λ-ERK dependent ETV5 overexpression transcriptionally activates SATB homoebox 2 to inhibit B-cell differentiation and maturation (Fig. 3C2, upper) [109].

## Dysregulation of ETV5 expression

**Fig. 4:**
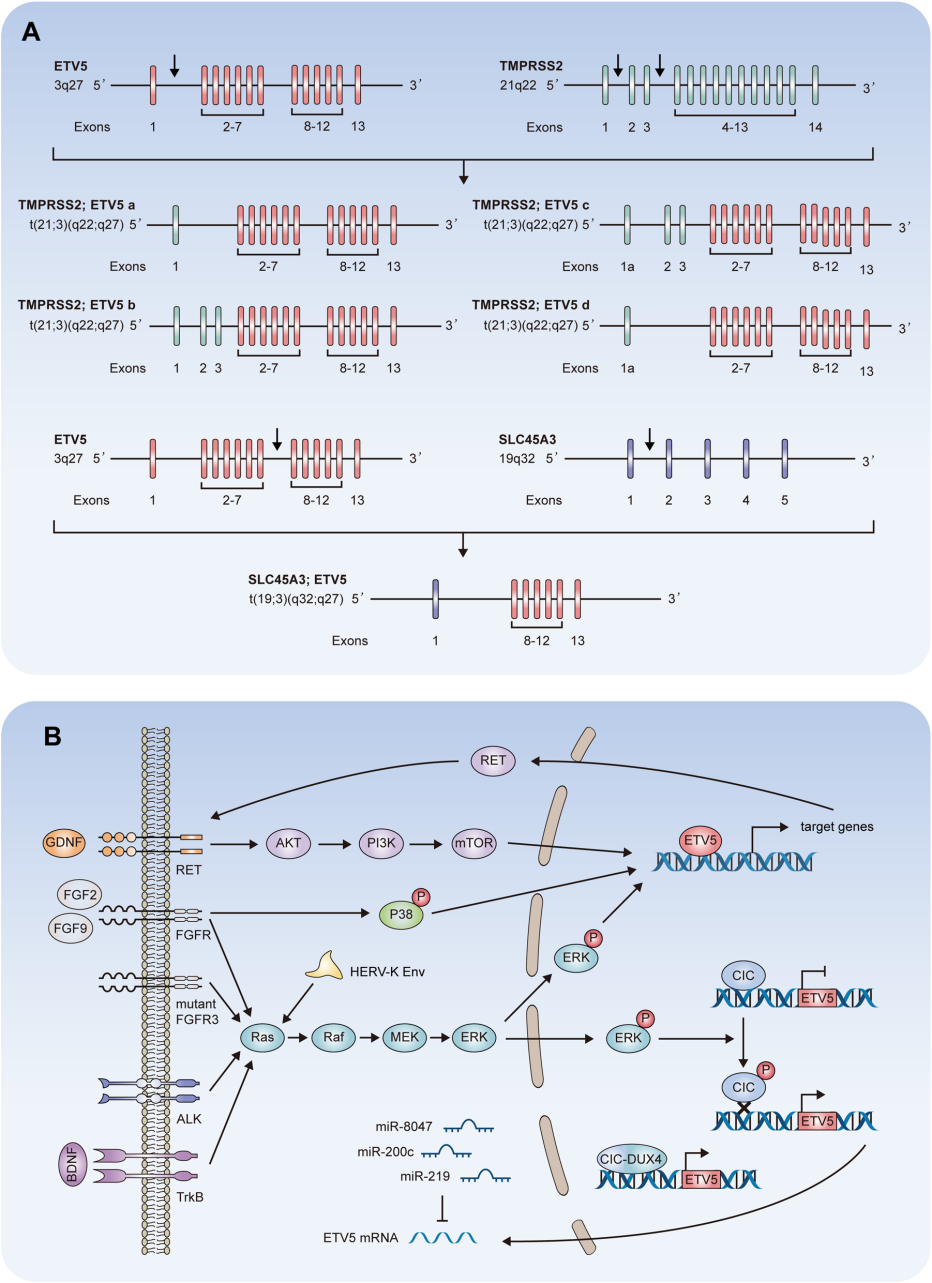
Dysregulation of ETV5 expression. **A** Fusion of TMPRSS2 and SLC45A3 with ETV5: the ETV5 gene has thirteen exons and exon 2 is the first coding exon. The breakpoints are located between exons 1 and 2 or exons 7 and 8. The TMPRSS2 gene contains two alternative first exons, 1 and 1a, followed by exons 2–14. Exon 1a is located approximately 1.5 kb downstream of exon (1) The hybrid transcripts yield four mRNAs in which TMPRSS2 sequences are fused to ETV5 exon (2) The SLC45A3 gene contains five exons. The SLC45A3:ETV5 transcript yields an mRNA in which SLC45A3 sequences are fused to ETV5 exon 8. **B** Crosstalk between ETV5 and molecular signaling pathways: ETV5 is activated through the ERK/MAPK, PI3K/AKT and p38/MAPK signaling pathways initiated by FGFR, GDNF/RET, TrkB and ALK. Activated ETV5 transcriptionally upregulates RET, which translocates to the cell membrane and activates the ERK/MAPK cascade. Phosphorylation of CIC by ERK/MAPK de-represses ETV5 transcription. CIC-DUX4 fusion acts as a transcriptional activator of ETV5. Human endogenous retrovirus-K (HERV-K) Env upregulates ETV5 in an ERK-dependent mechanism. ETV5 is downregulated by miR-8067, miR-219 and miR-200c

The expression of ETV5 was widely investigated in human tissues and multiple cancers. Aberrant ETV5 levels lead to developmental abnormalities of organs. Interestingly, ETV5 is mostly upregulated in cancers, with rarely being downregulated in some cases. The increased expression of ETV5 in cancers was found to be associated with clinicopathological features and patient prognosis, indicating its potential application as a diagnostic and prognostic biomarker (Table 1). Gene mutation, post-transcriptional modification and molecular signaling crosstalk all contribute to the dysregulation of ETV5 in cancers and physiological processes (Fig. 4).

**Table 1:** Oncogenic role of ETV5 in various cancer

Cancer type	Expression	Function	Clinical significance	Ref.
Bladder cancer	–	Proliferation	Positively correlated to TAZ	[112]
Breast cancer	–	EMT	Positively correlated to HER2, EGFR and histoprognostic grading Correlation with poor overall survival; a prognostic factor on overall survival in lymph node-positive patients	[18, 25, 127]
Chondrosarcoma	High expression in 105KC cell line	Bone resorption	–	[136]
Colorectal cancer	High expression in tumor tissues and TCGA and GEO dataset High expression in distal region of stage II/III colon cancer tissues	Proliferation, cell-cycle, angiogenesis, and drug resistance	Positively correlation to PDGF-BB, VEGFA and CCL2; negatively correlated to p21 Correlation with poor overall survival and disease-free survival	[16, 143, 146, 153]
Endometrial carcinomas	High expression in FIGO stage IB High expression at the invasive front of tumor tissues	EMT, migration, invasion, adhesion, oxidative stress	Positively correlation to NID1 in endometrial tumors; positively correlation to NID1 and NUPR1 at the invasion front of endometrial tumors	[17, 21]
Endometrioid endometrial carcinomas	High expression, especially in IA stage	Neoplastic transformation	–	[19, 26]
Esophageal squamous cell carcinoma	High expression in tumor tissues and TCGA and GEO dataset	Adhesion, migration, invasion	Correlation with poor overall survival and progression free survival	[22]
Gastric cancer	–	Migration, invasion	–	[15]
Glioblastoma	High expression in tumor tissues	Proliferation	Correlation with poor overall survival	[117, 122]
Oligodendrogliomas with CIC-mutant	High expression in tumor tissues and TCGA dataset	–	–	[118]
Lung cancer	–	Migration, invasion, tumor initiation	–	[13, 15]
Neuroblastoma	–	Proliferation, migration and invasion	Correlation with poor overall survival and progression free survival	[114, 115]
Ovarian cancer	High expression in tumor tissues	Cell-cycle, migration, invasion, angiogenesis and oxidative stress	Positively correlation to E-cadherin	[144, 147]
High-grade serous ovarian cancer	High expression in tumor tissues and cell lines	Proliferation, migration and invasion	Positively correlation to lncRNA CTBP1-DT and MAP3K3; correlation with poor overall survival and disease-free survival; have a prognostic value with recurrence and FIGO stage	[23]
Prostate cancer	–	Proliferation, migration, invasion	–	[128]
Synovial sarcoma	High expression in tumor tissues	Cell-cycle, chromatin structure	–	[38]
Thyroid cancer	High expression in tumor tissues and TCGA and GEPIA dataset	Proliferation, migration, invasion	Positively correlation to PIK3CA	[24, 34]

### Gene fusion

Researchers discovered rare 5′ fusions of ETV5 with TMPRSS2 and solute carrier family 45 member 3 (SLC45A3) in prostate cancer (Fig. 4A) [10]. Three different fusions of TMPRSS2 with ETV5 were discovered to date. In the TMPRSS2-ETV5a transcript, exon 1 of TMPRSS2 is fused with exon 2 of ETV5, which can produce the full-length ETV5 protein, while in the TMPRSS2-ETV5b transcript, the exons 1 to 3 of TMPRSS2 were fused with exon 2 of ETV5 [10]. The formation of TMPRSS2-ETV5c is interesting. In ETV5 outlier expression samples, about 1.5 kb downstream of the first exon in the TMPRSS2 reference sequence there was a novel 1st exon named 1a, which overlaps with a reported ETS sequence found in the tongue tumor library [110]. This novel 1st exon and exons 2 to 3 of TMPRSS2 were fused with exon 2 of ETV5, resulting in TMPRSS2-ETV5c. In addition, TMPRSS2-ETV5c produces a new fusion named TMPRSS2-ETV5d, characterized by the novel exon 1st fused to exon 2 of ETV5 [110]. In prostate cancer samples, SLC45A3 was also found to be a 5′ fusion partner, and exon 8 of ETV5 was fused to exon 1 of SLC45A3, resulting in ETV5 gene rearrangements [10]. Gene fusion protects ETV5 from protein degradation and thereby promotes oncogenic transformation in vivo [1, 9].

### Crosstalk between ETV5 and molecular signaling pathways

The transcriptional activity of ETV5 can be modulated by several signaling pathways, thereby affecting the expression of its target genes (Fig. 4B).

In kidneys, ETV5 is upregulated by GDNF-RET signaling. This is likely achieved through the phosphatidylinositol-3-kinase (PI3K) pathway, since PI3K inhibitors eliminated ETV5 expression (Fig. 4B, upper) [47]. FGF family activates the ERK-MAPK pathway and p38-MAPK pathway, which upregulate ETV5 in many tissues (Fig. 4B, middle). LY2874455 is a unique FGFR inhibitor with inhibitory effects on the downstream MAPK signaling pathways. ETV5 protein levels were decreased by LY2874455 treatment in small cell lung cancer cells [111]. In addition, ETV5 expression can be inhibited by BGJ398, a selective inhibitor of FGFR1-3 [38]. In SSCs, FGF2 and FGF9 upregulate ETV5 through p38-MAPK signaling [75]. During spermatogenesis, FGF2 upregulates ETV5 through the phosphorylation of mitogen-activated protein kinase 1, which in turn contributes to ERK-MAPK signal activation [73]. In bladder cancer cells, mutant FGFR3 increases ETV5 levels through an ERK-MAPK pathway-mediated mechanism [112]. Human endogenous retrovirus-K envelope proteins activate ERK-MAPK signaling to modify ETV5 expression, which confers oncogenic properties in breast epithelial cells [113]. Activated anaplastic lymphoma kinase (ALK) upregulates ETV5 via ERK-MAPK signaling [114]. The ALK-dependent upregulation of ETV5 promotes RET expression by direct binding to its promoter [115]. Through the ALK-ERK-ETV5-RET pathway, ETV5 acts as a link between activated RET and MEK/ERK signaling (Fig. 4B, lower) [114, 115]. In endometrial carcinoma, BDNF-TrkB upregulates ETV5 through ERK-MAPK signaling (Fig. 4B, lower) [21].

CIC, a downstream effector of the ERK-MAPK pathway, acts as a constitutive repressor of ETV5 (Fig. 4B, right) [116]. The CIC-ETV5 axis is an important modulator of biological behaviors in cancer cells. In glioblastoma, knock-down of CIC resulted in ETV5 upregulation, which was consist with the roles of CIC in the suppression of ETV5 [117]. The CIC-double homeobox 4 (DUX4) fusion oncoprotein retains CIC DNA-binding specificity but gains activating capacity to increase ETV5 transcription, which is consistent with the overexpression of ETV5 in CIC-mutant oligodendrogliomas [118, 119].

In chondrosarcoma, SOX9 enhances ETV5 promoter activity when co-transfect ETV5 promoter-reporter plasmid with SOX9 overexpressing lentivirus, but the specific binding sites and interaction mechanism remain to be determined [120]. In the model of cerulein induced pancreatitis, mice with conditional knockout of ETV5 exhibit delayed recovery from the inflammation and associate with decreased SOX9 expression in pancreas. In pancreatic ductal cells, the luciferase reporter assay demonstrates that upregulated ETV5 increases the activity of SOX9 promoter, indicating ETV5 might be an upstream regulator of SOX9 during pancreatitis [121]. While, SOX9^−/−^ mice undergo the cerulein induced pancreatitis maintain ETV5 expression [121].

### Post-transcriptional regulation mediated by microRNAs

In glioblastoma, low expression of ETV5 in tumor samples is associated with longer overall survival, which suggested that high expression of ETV5 might be a risk factor for glioblastoma [122]. Analysis of the ceRNA regulatory network associated with ETV5 indicated that ETV5 is regulated by miR-8067 in glioblastoma [122]. In human islets, the expression of ETV5 is correlated with miR-200c [123]. MiR-200c directly binds the 3′-untranslated terminal region (UTR) of ETV5 mRNA and reduces ETV5 expression, which decreased glucose-stimulated insulin secretion in type 2 diabetes [123]. Recently, it was demonstrated that miR-219 plays a critical role in central neuron system myelination and remyelination after injury [124]. MiR-219 directly targets ETV5 through a binding site in the coding region of ETV5 mRNA. During normal myelination, ETV5 is downregulated by miR-219 as a stage-specific target during oligodendrocyte differentiation [124]. In CD4^+^ Th cells, ETV5 acts as a functional target of miR-219a-5p through a binding site located in the 3′-UTR of ETV5 mRNA. The miR-219a-5p-ETV5 axis mediates Th1/Th17 cell differentiation during intestinal inflammation (Fig. 4B, middle lower) [103].

## ETV5 in cancer biology

**Fig. 5:**
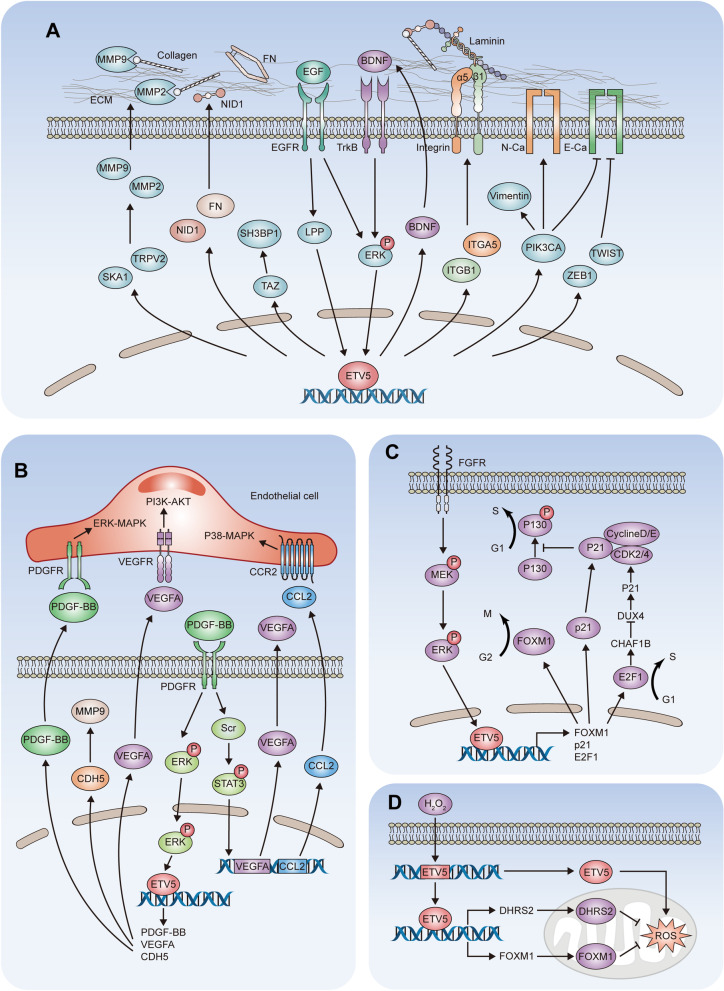
ETV5 in cancer biology. **A** ETV5 in cell mobility and EMT. ETV5 mediates the invasive phenotype through a BDNF-TrkB-ERK-ETV5-BDNF loop. epidermal growth factor receptor (EGFR)-LPP signaling promotes zinc finger E-Box binding homeobox factors 1 (ZEB1) expression through ETV5. ETV5 transcriptionally regulates the expression of TAFAZZIN (TAZ), twist family BHLH transcription factors 1 (TWIST), phosphatidylinositol-4,5-bisphosphate 3-kinase catalytic subunit alpha (PIK3CA), Vimentin, N-Cadherin and E-Cadherin. ETV5 upregulates NID1, fibronectin (FN), α5-integrin and β1-integrin to promote cancer cell adherence to laminin and collagens. ETV5 promotes spindle and kinetochore associated complex subunit 1 (SKA1) and transient receptor potential cation channel subfamily V member 2 (TRPV2) expression to upregulate MMP2 and MMP9, which degrade collagens to promote invasion through the ECM. **B** ETV5 in angiogenesis: ETV5 upregulates the mRNA expression of VEGFA, PDGF-BB and Cadherin 5 (CDH5). CDH5 promotes angiogenesis through the upregulation of MMP9. In an autocrine manner, PDGF-BB activates the ERK/MAPK and STAT3 to upregulate ETV5, VEGFA and CCL2. PDGF-BB, VEGFA and CCL2 activate ERK/MAPK, PI3K/AKT and p38/MAPK signaling in endothelial cells in a paracrine manner. **C** ETV5 in cell cycle: FGFs activate ETV5 via an ERK/MAPK-dependent pathway. Activated ETV5 promotes the G_1_/S and G_2_/M transition by enhancing E2F1 and FOXM1 transcription. Furthermore, ETV5 suppress the binding of p21 to CDK2/4 and Cyclin D/E, contributing to the phosphorylation of p130 and promoting the G_1_/S transition. Moreover, E2F1 induces chromatin assembly factor 1 subunit A/B (CHAF1B) to decrease DUX4 expression, which promotes p21 activity and cell cycle arrest. **D** ETV5 in oxidative stress: In Hec-1 A endometrial cancer, H_2_O_2_ promotes ETV5 and DHRS2 expression, resulting in increased ROS levels. In ovarian cancer, ETV5 upregulates the expression of FOXM1, which protects cells from oxidative stress. *EGF* epidermal growth factor, *CCR2* C-C motif chemokine receptor 2, *Src* nonreceptor tyrosine kinase

Cancer progression is a complex and coordinated process that requires a multitude of genetic events and oncological transition of cell biology [125]. ETV5 activation contributes to many oncogenic and metastatic features of cancer cells, including proliferation, mobility, invasiveness, angiogenesis, and drug resistance (Fig. 5).

### Cell mobility and EMT

The epithelial–mesenchymal transition (EMT) is a crucial cellular program that facilitates the loss of epithelial characteristics and the acquisition of mesenchymal features, with increases the mobility of adherent epithelial cells and contributes to the dissemination of tumor cells (Fig. 5A) [126]. ETV5 altered chromatin accessibility and regulates the expression of a subset of EMT-related genes, contributing to the TGF-β-induced pro-tumorigenic phenotype of mammary gland epithelial cells [127]. In prostate cancer, ETV5 promoted the EMT and cell migration through transcriptional regulation of TAFAZZIN expression [128]. In thyroid cancer cells, 17b-estradiol increased cell viability and ETV5 expression [129]. Silencing of ETV5 resulted in higher expression of E-cadherin as well as lower expression of N-cadherin and vimentin, which was mediated by the interaction with the promoter of phosphatidylinositol-4,5-bisphosphate 3-kinase catalytic subunit alpha [129]. In thyroid cancer, ETV5 promoted the mesenchymal morphology of cancer cells by regulating the mRNA levels of twist family BHLH transcription factors 1 (TWIST1) and snail family transcriptional repressors 1 (SNAI1). ETV5 directly bound to the promoter of TWIST1 to control its expression. However, the enrichment of ETV5 was not observed in the SNAI1 promoter, which indicating that ETV5 may indirectly regulating SNAI1 expression [130]. In lung cancer cell lines, LIM domain containing preferred translocation partner in lipoma (LPP) and ETV5 were found to act cooperatively to inhibit N-cadherin through the transcriptional regulation of MMP15 [131]. In endometrial carcinoma, BDNF/TrkB-ERK was found to upregulate ETV5, which mediates the invasive phenotype of cancer cells [21]. Upon epidermal growth factor stimulation, LPP translocated into the nucleus and induced ETV5 expression, which transcriptionally regulated zinc finger E-Box binding homeobox factors 1 and then repressed E-Cadherin expression [17]. Overexpression of ETV5 is associated with elevated levels of fibronectin, α5 integrin, β1 integrin, and N-Cadherin, as well as diminished levels of Cyclin D1 and β-Catenin, promoting a more invasive phenotype of endometrial carcinoma [17].

Cell-substrate interactions are mainly mediated by the extracellular matrix (ECM), which contributes to cell mobility during the EMT [126]. Nidogen 1 (NID1), a basement membrane glycoprotein, stabilizes the basement membrane by forming a non-covalent high-affinity bridge by binding to laminin and type IV collagen [132]. ETV5 was fond to regulate NID1 directly at the transcriptional level by binding to its proximal promoter region. The upregulation of NID1 promoted the adhesion of cancer cells to laminin, thus promoting cell invasion and migration [133]. MMPs are the enzymes responsible for the degradation of the ECM [134]. ETV5 was found to promote the transcription of spindle and kinetochore associated complex subunit 1 and transient receptor potential cation channel subfamily V member 2, both of which were found to contribute to MMP2 and MMP9 expression in esophageal cancer [22]. ETV5 also directly transcriptional regulates MMP2 expression and gelatinase activity, which confer an invasive phenotype on cancer cells (Fig. 5A) [15, 135, 136].

Now, there is substantial evidence that ovarian cancers mostly arise from the fallopian tube epithelia, which has stromal characteristics and experiences epithelial differentiation during neoplastic progression [137, 138]. Therefore, ovarian cancer is characterized by increased expression of epithelial markers rather than following the classical model of the EMT, the process of which is different from other epithelial tumors. In ovarian cancer, ETV5 was found to promote cell proliferation, migration and invasion through the regulation of lncRNA CTBP1-DT [139]. Cancer cells with downregulated ETV5 exhibited reduced adhesion to collagen type I, collagen type IV, fibronectin, and laminin [140]. ETV5 induced the expression of ZO-1 and E-cadherin as well as repressing the expression of N-cadherin, contributing to increased cell–cell interactions [140].

### Angiogenesis

Platelet-derived growth factor beta polypeptide b (PDGF-BB), a key regulator of blood vessel formation, can activate nonreceptor tyrosine kinase and STAT3 to trigger the production of angiogenic factors, such as vascular endothelial growth factor A (VEGFA) and FGF [141]. In colorectal cancer (CRC), ETV5 was found to upregulate PDGF-BB expression by binding to the promoter of PDGF-BB. The upregulated PDGF-BB then interacts with its receptors to activate ERK-ETV5 signaling, forming a positive feedforward loop in ETV5-mediated CRC angiogenesis [16]. It was demonstrated that C-C motif chemokine ligand 2 (CCL2) could directly stimulate angiogenesis by binding to C-C motif chemokine receptor 2 on vascular endothelial cells [142]. In CRC, ETV5 was found to activate VEGFA expression by directly binding its promoter region, which induced CCL2 secretion via STAT3, acting as a TF of CCL2 [143]. ETV5-induced secretion of VEGFA and CCL2 was found to activate PI3K/AKT and p38/MAPK signaling in human umbilical vein endothelial cells, which promoted angiogenesis in CRC [143]. ETV5 was found to promote angiogenesis via transcriptional activation of Cadherin 5, which upregulated MMP9 expression in ovarian cancer cell lines (Fig. 5B) [144].

### Cell growth and cell cycle transition

Abnormal activity of the cell cycle is a hallmark of tumors, resulting from the aberrant activation of cell-cycle proteins [145]. Upregulation of ETV5 was found to promote the G1-S phase transition by directly suppressing the transcriptional activity of cyclin-dependent kinase inhibitor 1 A (CDKN1A) (Fig. 5C) [38, 146]. ETV5 was also found to suppress p21 bound to CDK2/4, which phosphorylates p130 and promotes G1-S phase transition [146]. In OV90 ovarian cancer cells, ETV5 was found to upregulate fork head box M1 (FOXM1) expression by binding to its promoter region, leading to the increased transcription of cell-cycle genes involved in G_1_–S and G_2_–M progression, including cyclin D, cyclin B, cell division cycle 25 A, cell division cycle 25B, CDKN1A and cyclin-dependent kinase inhibitor 1B [147]. Synovial sarcoma fusion SS18-SSX1 was found to activate FGF/FGFR signaling by recruiting FGFR2. The activated signaling increased the expression of ETV5, which promoted cell cycle progression by directly binding the E2F transcription factor 1 (E2F1) promoter [38]. E2F1 induced the expression of chromatin assembly factor 1 subunit A/B, leading to decreased expression of DUX4, which contributed to cell cycle arrest by cyclin-dependent kinase (Fig. 5C) [38, 148].

### Oxidative stress

In Hec-1 A endometrial cancer cells, ETV5 overexpression resulted in increased intracellular levels of reactive oxygen species (ROS) and mitochondrial dehydrogenase 2 (DHRS2) protein levels. Exposing Hec-1 A cells to H_2_O_2_ can increase the protein expression of ETV5 and DHRS2. At the same time, by directly binding to the promoter region of DHRS2, ETV5 might play an important role in protecting mitochondria from the cytotoxic effect of reactive α-dicarbonyls [149]. In ovarian cancer, OV90 cells with downregulation of ETV5 exhibited an increase of ROS production. When exposed to exogenous H_2_O_2_, the protein levels of ETV5 and FOXM1 increased. Moreover, these changes are more apparent in OV90 cells with low endogenous ETV5 levels, which suggests a potential role of ETV5 in protecting cells from oxidative stress via the upregulation of FOXM1 (Fig. 5D) [147].

### Drug resistance

The majority of ovarian cancer patients have an initial response to paclitaxel (PTX), but a significant proportion of patients eventually develops chemoresistance, which remains an obstacle in clinical practice [150]. In ovarian cancer cells, ETV5 was found to contribute to the acquisitions of resistance to PTX, and the level of ETV5 protein is mediating by the miR-1307-CIC axis [151]. In colon cancer, 5-fluorouracil (5-FU)-based adjuvant chemotherapy is recommended for high-risk stage II patients as well as stage III patients, but its application is hampered by considerable toxicity and economic cost [152]. ETV5 expression is associated with the overall survival of patients treated with 5-FU-based adjuvant chemotherapy and affects the survival response of 5-FU treated stage III patients, suggesting that ETV5 can be used as a predictive biomarker for 5-FU-based adjuvant chemotherapy responses in stage II/III patients [153]. Cetuximab (CTX), a monoclonal antibody against epidermal growth factor receptor, is currently used in patients with wild-type (WT) KRAS [154]. However, almost half of the patients with WT KRAS failed to respond to this treatment [155]. By analyzing the gene expression data of CRC patients treated with CTX monotherapy, ETV5 was found be upregulated in CTX-resistant tumors [156]. At the same time, knockdown of ETV5 increased CTX sensitivity and erlotinib susceptibility in KRAS WT cell lines, suggesting that ETV5 might be a potential target for overcoming CTX resistance in CRC [156]. BRAF is a serine/threonine kinase that harbors activating mutations in 60% of melanomas [157]. Despite initial clinical responses to BRAF inhibitors, patients frequently develop drug resistance. The JUN family TFs and ETV5 are essential regulators of CDK6, which together mediate resistance to BRAF inhibitors in melanoma cells [158]. Based on the gene expression profiles correlated to drug responses, ETV5 was identified as a biomarker of tumor sensitivity to the MEK inhibitors cobimetinib and selumetinib [159, 160].

## Conclusions and perspectives

**Fig. 6:**
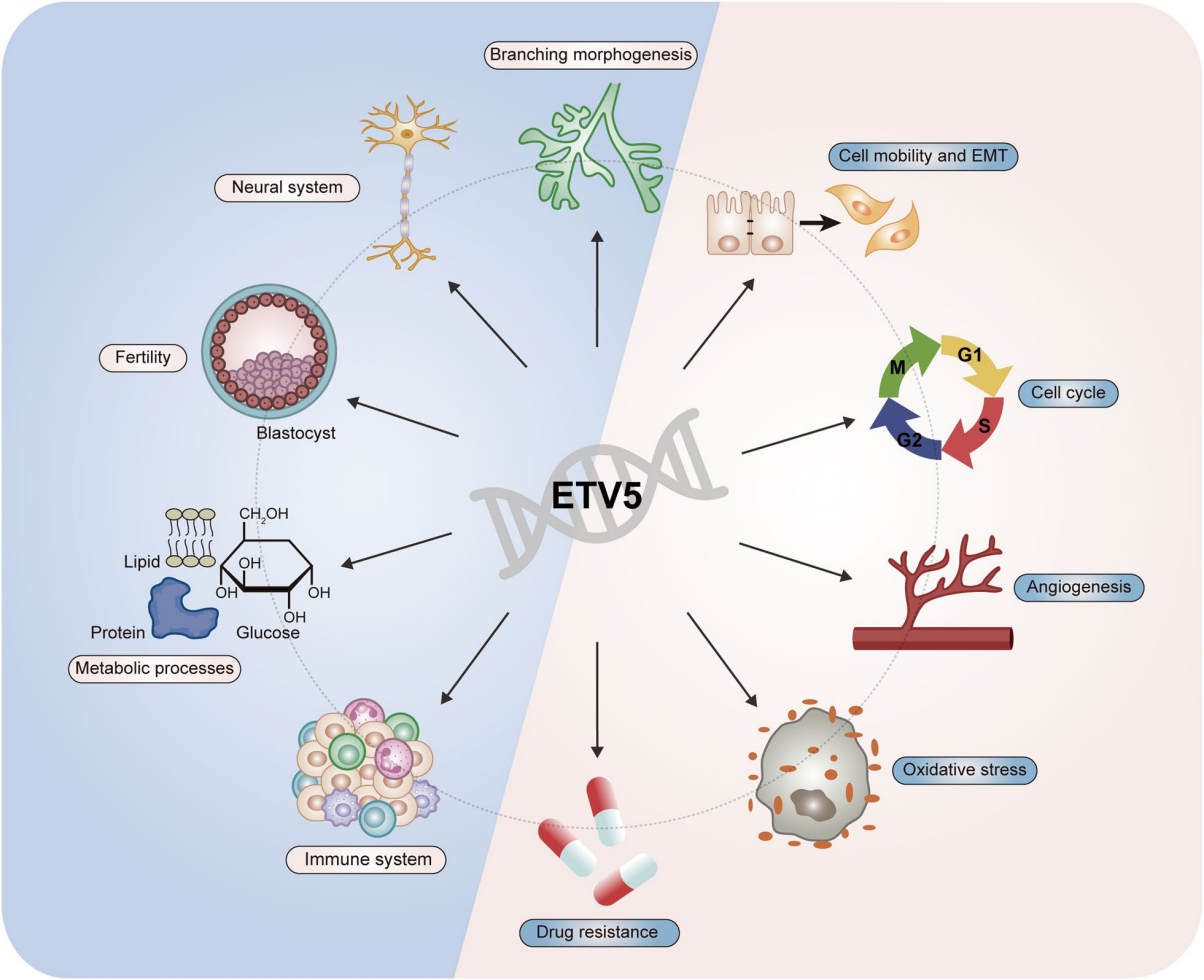
ETV5 in the regulation of physiological activities and cancer hallmarks. ETV5 regulates a larger number of physiological activities and cancer hallmarks, including branching morphogenesis, neural system development, fertility and embryonic development, metabolic processes, immune system function, cell mobility and EMT, angiogenesis, cell growth and cell cycle transition, oxidative stress and drug resistance

ETV5 acts as a crucial regulator of biological processes by regulating the transcriptional activity of multiple genes. The dysregulation of ETV5 contributes to progression of many benign disorders and cancers. To date, ETV5 was found to participate in physiological processes of kidney and lung development, embryonic survival, energy balance, neuron growth, and T cells differentiation. From the perspective of oncology, numerous studies revealed its role and molecular mechanism as an oncogenic TF, indicating that it may be a promising prognostic and diagnostic marker for cancers (Fig. 6).

Despite these tremendous advances, a number of questions remain unanswered. Most of the current knowledge on the molecular activities of ETV5 were obtained from studies on solid tumors, and little is known on the roles of ETV5 in hematological malignancies. Studies have shown that ETV5 contributes to the normal development of the immune system. However, the possible involvement of ETV5 in onco-immunology still remains to be investigated. Post-translational modifications influence the expression, degradation and transcriptional activity of ETV5. The specific SUMOylation and MAPK-dependent phosphorylation sites and the underlying mechanisms need to be studied further, as these may reveal novel druggable targets for ETV5-based cancer treatment.

ETV5 is frequently upregulated in human cancers and induces tumorigenesis via multiple mechanisms. Thus, ETV5 inhibition may produce pleiotropic effects. Ablation of ETV5 synergizes with chemotherapy in reducing tumor growth and affecting the survival response of patients. Therefore, ETV5 may be a valuable therapeutic target for the treatment of human malignancies. ETV5 is a well-known downstream target gene of the ERK pathway and acts as a biomarker of tumor sensitivity to MEK and BRAF inhibitors. Pharmaceutical research focusing on the inhibition of the MAPK pathway might identify novel inhibitors of ETV5 as a basis for anti-cancer drug design in the future. ETV5 depends on the recruitment of co-activators or suppressor to activate downstream genes, which indicates that these interacting proteins could also be promising targets for blocking the carcinogenic effect of ETV5. The half-life of ETV5 protein is regulated by SUMOylating and ubiquitination, so interrupting these post-translational modifications is an additional strategy for inhibiting ETV5 function. As ETV5 is considered an undruggable transcription factor, many questions must still be addressed in order to develop efficient ETV5-based therapeutic strategies. Nevertheless, the great clinical application potential of ETV5-based therapies merits further studies in the future.

## Data Availability

Not applicable.

## Abbreviations

AA: Amino acids
ALK: Anaplastic lymphoma
aPKC: Atypical protein kinase C
ATI: Alveolar type I
ATII: Alveolar type II
BAX: BCL2 associated X
BCL6B: BCL6B transcription repressor
BDNF: Brain-derived neurotrophic factor
BRAF: B-Raf proto-oncogene
CCL2: C-C motif chemokine ligand 2
CCL9: C-C motif chemokine ligand 9
CDK: Cyclin dependent kinase
CDKN1A: Cyclin dependent kinase inhibitor 1 A
CIC: Capicua transcriptional repressor
CID: C-terminal inhibitory domain
COP1: Constitutive photomorphogenic 1
CRC: Colorectal cancer
CRL4: Cul4-RING ubiquitin ligase
CTX: Cetuximab
CXCL12: C-X-C motif chemokine ligand 12
CXCR4: C-X-C motif chemokine receptor 4
DBD: DNA-binding domain
DDB1: DNA damage-binding protein 1
DET1: De-etiolated 1
DHRS2: Dehydrogenase 2
DRG: Dorsal root ganglion
DUX4: Double homeobox 4
E2F1: E2F transcription factor 1
ECM: Extracellular matrix
EMT: Epithelial–mesenchymal transition
ERM: ETS-related molecule
ESCs: Embryonic stem cells
ETS: E26 transformation-specific
ETV5: E26 transformation-specific transcription variant 5
FGF: Fibroblast growth factor
FGFR2: Fibroblast growth factor receptor 2
T_FH_: Follicular helper CD4^+^ T
FOXM1: Fork head box M1
GABA: γ-Aminobutyric acid
GATA6: GATA binding protein 6
GDNF: Glial cell line derived neurotrophic factor
GOAT: Ghrelin-*O*-acyl-transferase enzyme
HPA: Hypothalamic-pituitary-adrenocortical
IL: Interleukin
LPP: LIM domain containing preferred translocation partner in lipoma
MAPK: Mitogen-activated protein kinase 3
MMP: Matrix metallopeptidase
NEUROG2: Neurogenin 2
NGF: Nerve growth factor
NID: N-terminal inhibitory domain
NID1: Nidogen 1
NPCs: Neural progenitor cells
NRD: Negative regulatory domain
PDGF-BB: Platelet-derived growth factor beta polypeptide b
PI3K: Phosphatidylinositol-3-kinase
PKA: Protein kinase A
PPARs: Peroxisome proliferator-activated receptors
PPREs: PPAR elements
PS1: Presenilin 1
PTX: Paclitaxel
RET: Ret proto-oncogene
ROS: Reactive oxygen species
SDF-1: Stromal cell-derived factor 1
SHH: Sonic hedgehog signaling molecule
SLC45A3: Solute carrier family 45 member 3
SNAI1: Snail family transcriptional repressors 1
SOX9: Sex-determining gene SRY-box9
SSCs: Spermatogonial stem cells
STAT: Signal transducer and activator of transcription
SUMO: Small ubiquitin-related modifier
TAD: Terminal acidic transactivation domain
TERTp: Telomerase reverse transcriptase promoter
TF: Transcription factor
Th: T helper
TMPRSS2: Transmembrane protease serine 2
Trk: Tropomyosin receptor kinase
Trm: Tissue-resident memory T
TWIST1: Twist family BHLH transcription factors 1
UB: Ureteric bud
UTR: Untranslated region
VEGFA: Vascular endothelial growth factor A
WT: Wild-type
5-FU: 5-Fluorouracil
